# The optimal use of computer aided detection to find low prevalence cancers

**DOI:** 10.1186/s41235-022-00361-1

**Published:** 2022-02-04

**Authors:** Melina A. Kunar

**Affiliations:** Department of Psychology, The University of Warwick, Coventry, CV4 7AL UK

**Keywords:** Low prevalence, Mammogram, Visual search, Computer aided detection (CAD), Decision-making

## Abstract

People miss a high proportion of targets that only appear rarely. This low prevalence (LP) effect has implications for applied search tasks such as the clinical reading of mammograms. Computer aided detection (CAD) has been used to help radiologists search mammograms by highlighting areas likely to contain a cancer. Previous research has found a benefit in search when CAD cues were correct but a cost to search when CAD cues were incorrect. The current research investigated whether there is an optimal way to present CAD to ensure low error rates when CAD is both correct and incorrect. Experiment 1 compared an automatic condition, where CAD appeared simultaneously with the display to an interactive condition, where participants could choose to use CAD. Experiment 2 compared the automatic condition to a confirm condition, where participants searched the display first before being shown the CAD cues. The results showed that miss errors were reduced overall in the confirm condition, with no cost to false alarms. Furthermore, having CAD be interactive, resulted in a low uptake where it was only used in 34% of trials. The results showed that the presentation mode of CAD can affect decision-making in LP search.

## Introduction

Visual search is an important part of our everyday life, whether it is searching for a mobile phone in a living room, a child in a playground, or car in a car park. Some visual search tasks have significant implications for our health and safety. For example, a baggage screener searching through x-rays for a prohibited item or radiologists searching through mammograms for a cancer. These latter searches are made all the more difficult given that the targets only appear rarely (e.g. cancers typically appear in fewer than 1% of cases, Gur et al., 2003) and that search for a low prevalence item leads to a large proportion of miss errors (Wolfe et al., 2005). Given the importance of finding a rare mass in radiology and the serious implications of missing a cancer, it is critical to find ways to help detection of a low prevalence target. One method to help with this is computer aided detection.

Computer aided detection (CAD) uses computer algorithms to identify areas of interest within a mammogram and mark them for radiologists to inspect, with the aim to help readers better detect a cancer (Castellino, 2005; Gilbert et al., 2008; Lehman et al., 2015). The use of CAD is available globally, with some countries using CAD systems more than others (e.g. Guerriero et al., 2011; Houssami et al., 2009; Lehman et al., 2015; Sato et al., 2014). Research into how best to use CAD is vital given that there has been a large investment into its development to help radiologists search mammograms (estimated to cost over $400 million a year, Lehman et al., 2015). However, at present, research has shown that CAD technology does not measure up to expectation with little benefit in cancer detection (e.g. Fenton et al., 2007, 2011; Lehman et al., 2015). One of the issues is that CAD systems are typically tested using enriched sets of mammograms where cancer prevalence is high. However, in a clinical setting the prevalence of a cancer is much lower (Horowitz, 2017). This leads to problems as search performance at high prevalence is not necessarily representative of search performance at low prevalence. Furthermore, there is little research into how to best present CAD to readers for optimal reading.

### Why is it important to consider prevalence rates in search?

Kundel (1982) was one of the first researchers to highlight the issue of prevalence in the medical field and noted that the prevalence of a disease needed to be considered when reporting observational studies in radiology and the performance of radiology image systems. In the clinical field, Egglin and Feinstein (1996) and Ethell and Manning (2001) found that prevalence rates affected detection of pulmonary emboli and wrist fractures, respectively, with lower detection rates at lower prevalence. Wolfe et al. (2005) investigated this effect in the laboratory where they designed a study in which participants searched for a low prevalence target in a visual search task. Participants were asked people to detect a target that could appear 50%, 10% and 1% of the time. With this reduction in prevalence rates there was a marked increase in the number of targets that were missed (from 7 to 16% and 30%, respectively). This increase in miss errors when the target is rare is known as the low prevalence (LP) effect and has been replicated multiple times (e.g. Kunar et al., 2010, 2021; Mitroff & Biggs, 2014; Rich et al., 2008; Russell & Kunar, 2012; Van Wert et al., 2009; Wolfe et al., 2007).

Several accounts have been proposed for the LP Effect. Fleck and Mitroff (2007) suggested that the LP Effect was due to a motor bias, whereby at low prevalence there was an increased proportion of motor errors, due to the propensity of participants pressing the ‘target absent’ key. However it has been shown that, this theory cannot account for the full LP effect as, even with the removal of motor-errors, participants missed a larger proportion of targets at LP compared to when targets had a high prevalence (Van Wert et al., 2009; Kunar et al., 2010, 2017a, 2017b, 2021; Russell & Kunar, 2012; Rich et al., 2008; see also Horowitz, 2017 for a discussion).

Wolfe and Van Wert (2010) proposed a multiple decision model (MDM), which suggested that the LP effect occurred due to two reasons. First at LP, the quitting threshold for when a participant decides to stop searching is reduced so that people make a response before they search the display sufficiently. Evidence in support of this comes from Rich et al. (2008) who found that people made fewer eye movements, and failed to fixate the target more often, at LP compared to when the target had a High Prevalence (HP, see also Peltier & Becker, 2016). Second, the MDM proposed that under LP conditions, people showed a criterion shift, where responses become more conservative. That is at LP, people were less willing and needed more evidence before responding that a target was present. This has been supported from studies using Signal Detection Theory (SDT, Green & Swets, 1967; Macmillan & Creelman, 2005) where a shift in response bias (as measured by *c*) has been observed at LP (Wolfe et al., 2007; see also Horowitz, 2017; Drew et al., 2020; Kunar et al., 2021; Russell & Kunar, 2012; Van Wert et al., 2009; Wolfe & Van Wert, 2010).

The majority of LP studies have been laboratory studies (e.g. Drew et al., 2020; Fleck & Mitroff, 2007; Kunar et al., 2021; Mitroff & Biggs, 2014; Rich et al., 2008; Russell & Kunar, 2012; Wolfe et al., 2005, 2007). However, Evans et al. (2013a, 2013b) found a similar effect occurred in a clinical setting, in which they embedded a mammogram known to contain a cancer into a medical reading procedure. It was found that trained readers missed this cancer 30% of the time, showing that even in a clinical setting, readers are prone to miss rare targets. Other studies have investigated ways to improve LP search (e.g. Wolfe et al., 2007). Kunar et al. (2021) found that having two observers search the same mammogram led to a reduction in miss errors (see also Wolfe et al., 2007). If two readers read the same mammogram in the same room at the same time, target detection was improved due to an improvement in sensitivity (as measured by SDT, using *A*′). However, if two observers read the same display independently (e.g. in separate rooms) then target detection was improved, as the response bias shift, typically observed at LP, was reduced. Although double reading leads to improved LP search and was previously deemed to be a cost-effective procedure to run in the UK, this practice may not be sustainable in the future as the population of women that need to be screened increases (Guerriero et al., 2011). Furthermore, double reading procedures are expensive with double the number of radiologists needed and may be difficult to sustain with an aging population (James et al., 2010). In response to this rise in demand, computer aided detection has been proposed as a way to simulate double reading procedures, in which CAD acts as the second reader without the increasing expenditure of human labour in terms of both time and financial costs (Azavedo et al., 2012).

### The benefits and costs of computer aided detection

CAD has been approved for use in mammography by the Food and Drug Administration (FDA) in the USA, with the aim to improve work-flow and reduce demands on radiologists and trained readers (Castellino, 2005; Gilbert et al., 2008). It has been evaluated in the clinical field either by the use of Randomised Control Trials (RCTs) or by recruiting radiologists or other trained readers to read mammograms in an observational setting (e.g. Gilbert et al., 2008; Hupse et al., 2013; Freer and Ulissey, 2001). RCTs have the benefit in that they can evaluate CAD in a real clinical environment. However, they are disadvantaged as there is no way to know the true miss errors that occur, as the radiologist, by definition will be unaware that they have missed a potential abnormality (unless a mass presents at a later scan or the woman becomes symptomatic at a later date). Furthermore, RCTs often involve lengthy periods of data collection (e.g. one RCT investigating CAD versus a double reading procedure took over 7 years for data collection, Gilbert et al., 2008) and scientists are also ethically limited in what can be manipulated in the normal clinical reading procedure to avoid potential disruption to a patient’s care.

In contrast, observational studies using radiologists or trained readers have the benefit of being able to test a greater range of CAD conditions by using ‘truth cases’ (i.e., mammograms that are known in advance to contain a cancer or not). Here, different reading conditions can be scientifically manipulated and investigated, without the same ethical concerns needed in real-life reading where patient care is at stake. However, these studies are limited by the time-constraints of radiologists and trained readers, so that (1) studies may be under-powered due to the low availability of readers (in some cases as few as 2 or 3, e.g. Freer and Ulissey, 2001) and (2) they may be tested under conditions where the target has a high prevalence (given that low prevalence data collection is lengthy and highly time-consuming, typically requiring thousands of trials). These differences in procedures in observational studies may also affect the way that radiologists respond, causing them to either under or over-estimate the number of cases that need to be recalled (Castellino, 2005).

In response, Kunar et al. (2017a, 2017b) developed a laboratory based, mammogram-reading procedure to complement RCTs and observational studies investigating CAD. In this study naïve readers were recruited and trained to search for LP targets, with the premise that the underlying mechanisms within the ‘human visual search engine’ are universal across experts and non-expert searchers (Wolfe et al., 2016). These procedures had the advantage of being able to recruit enough participants for sufficient experimental power in LP conditions. Kunar et al. (2017a, 2017b) found that having a valid CAD cue led to improved target detection compared to when no CAD cue was presented. However, miss errors greatly increased on trials when the target was present but the CAD cue was incorrect (i.e. it marked an area that did not contain a cancer) or was not presented (a cancer was present but had not been flagged up by a CAD cue). Kunar et al. (2017a, 2017b) proposed an over-reliance hypothesis whereby participants became over-dependent on CAD, rather than rely on their own judgements, affecting their capacity to find a target when CAD technology failed (see also Russell & Kunar, 2012 and Drew et al., 2020, who found similar evidence using eye movements).

The above research shows that there are both benefits and costs of using CAD and that optimal use of this technology depends on its human–computer interaction. Given that co-operation between human observers and CAD technology is vital, it is also important to examine how best to present CAD to maximise its benefit. In current US clinical practice, readers are required by the FDA to view the image alone first and then view the image with the use of CAD (Castellino, 2005). This reading procedure has its benefits. Drew et al. (2020) investigated two CAD systems using a visual search task where participants were asked to search for a letter T among distractor Ls (prevalence rate of 10%). In one of their experiments, CAD cues were presented automatically alongside the search display. In a different experiment, participants used the CAD cue interactively, in which they clicked on an area of the display which would then present a CAD recommendation. Target prevalence was also manipulated to contain both high and low prevalence conditions. From these experiments it was shown that having an LP target exacerbated the costs of an incorrect CAD cue compared to HP (see also Kunar et al., 2017a, 2017b), however having the CAD cue be interactive mitigated these costs. Please note, that this benefit was in relation to a condition where people were never shown a CAD cue rather than in relation to one where participants were automatically shown the CAD cue (which Drew et al., 2020, did not examine). Furthermore, Hupse et al. (2013) compared CAD prompts that were shown automatically to a condition in which readers could interactively use CAD. They also found the use of interactive CAD to be a more effective tool for detecting masses in mammograms.

These studies *indicate* that under LP conditions there is a benefit in using CAD interactively. However, there are some limits to this research which means that this hypothesis has not been directly tested. As mentioned above, although Drew et al. (2020) investigated two different ways of presenting CAD, these presentation methods were never directly analysed or compared to determine which presentation method led to fewer miss errors or false alarms at Low Prevalence (as this was outside the remit of their research question). Instead, each presentation method was compared to a condition where no CAD cues were used. Therefore, from Drew et al. (2020) the optimal presentation method of presenting CAD cannot be established. Direct comparisons of CAD presentation mode were made by Hupse et al. (2013). However, they used an experimental design in which the target had a high prevalence (which we know has different search mechanisms to LP, Wolfe & Van Wert, 2010, Horowitz, 2017) and some of their mammograms were repeated to the readers across experimental sessions: a procedure known in the visual search literature to improve people’s search performance (Chun & Jiang, 1998).

Despite the FDA’s requirement for readers to first view the medical image alone before the use of CAD, other researchers have suggested there is a cost to this viewing method. For example, Du-Crow et al. (2019) have suggested that viewing the image alone first, before the presentation of CAD may lead readers to feel a false sense of security (or ‘safety net’) as the expectation is that CAD will highlight any potential abnormalities that have been missed. Du-Crow et al. (2019) found supporting evidence of this using eye movements, which showed that on the initial (pre-CAD) search of an image, the percentage of image covered (as measured by the area surrounding fixations) was less than when people were asked to search a condition with no CAD.

In summary, there is no clear consensus, of the optimal way to present CAD when the target has a low prevalence. We know that presenting CAD concurrently with the search display, leads to an over-reliance on the CAD cues (Drew et al., 2012; Kunar et al., 2017a, 2017b). Does changing the presentation mode of CAD lessen this over-reliance? One reason for this over-confidence in CAD could be that CAD markers acts as ‘attention grabbing’ bottom-up attentional cues (e.g. Drew et al., 2020; Theeuwes, 2004). Given their salience, participants may not be able to help but attend these cues, if they appear simultaneously with the display, which might affect their judgements (see Kunar et al., 2017a, 2017b). Therefore, having the CAD cue appear at a later stage, after the mammogram has already been searched, may alleviate this issue: as the salient markers do not appear on first reading they do not affect initial judgements (this is especially important as early and initial processing of the image is an important factor that enables experts to determine the presence of a cancer, Evans et al., 2013a, 2013b). This was investigated across two experiments, in which CAD presentation modes were directly compared. In Experiment 1, CAD cues were either presented automatically alongside the mammogram (replicating conditions of Kunar et al., 2017a, 2017b and Drew et al., 2012) or presented interactively, where participants could *choose* to have the CAD presented after the initial display, should they want verification (the interactive condition). Experiment 2 compared CAD presentation in conditions where CAD was presented automatically with the display to when CAD was *always* presented after initial reading of the display (confirm condition). It was predicted that having people search the display initially before CAD would lead to fewer false alarms and miss errors when the CAD cue was incorrect compared to when CAD appeared automatically. This is because participants’ judgements would not be affected by the presence of a salient CAD cue in initial reading. However, after people had viewed CAD then the proportion of targets that were found would be equivalent when the CAD cue accurately predicted the target location.

Of final note, these experiments were also used to determine the behavioural preference of people to use CAD when they were given a choice. The interactive condition (Experiment 1) would be identical to the confirm condition (Experiment 2) if people made the choice to use the CAD cue. That is, CAD would only be effective in the interactive condition if there was a behavioural preference to use this for the majority of trials. As CAD has been proposed to act as double reader, in place of a human observer (Azavedo et al., 2012) then it is essential that people chose to interact with it. If people prefer to opt out of using CAD in the interactive condition then this behavioural preference has implications for the efficacy of CAD use overall. This was investigated in Experiment 1.

## Experiment 1

### Method

#### Participants

Twenty participants (*M* = 19.2 years, 11 female, 9 male) took part in Experiment 1. In all experiments, participants were recruited from the University of Warwick participant pool, had no prior training in reading mammograms and were paid for their time. All participants had normal or corrected-to-normal vision. Ethical approval for all studies was granted by the Humanities and Social Sciences Research Ethics Committee at the University of Warwick. Participant numbers were determined in advance based on previous research (e.g., Drew et al., 2012; Kunar et al., 2017a, 2017b; Wolfe et al., 2007). A power analysis calculated using G*Power (*F*-tests, effect size = 0.25, alpha = 0.05, see Faul et al., 2007) showed that the minimum number of participants needed to achieve a power of 0.8, for each experiment was 12 (based on the trial numbers in each condition). Therefore, we would expect that testing 20 participants for each of the experiments would provide ample power to detect significant effects, if present.

#### Stimuli and procedure

The experiment was programmed using BlitzMax and presented on a PC. The mammogram images were taken from the selection of ‘normal’ mammograms (those not containing a cancer) of the Digital Database for Screening Mammography (DDSM) database (Heath et al., 1998, 2001). All images were selected from the database at random. Images were presented in the centre of the display and subtended approximately 11 degrees by 19 degrees at a viewing distance of 57 cm (although the individual size of each image varied because they were real mammograms).^1^ For target present trials cancerous mass images were selected at random from four of the cancer cases on the DDSM. These cancers were then transposed onto mammograms that previously contained no cancer using imaging editing software so that each image contained one cancer (each cancer appeared equally as often throughout the experiment). The cancer could appear on any area of the breast tissue again chosen at random (mimicking conditions in a clinical setting), provided that it was clearly distinguishable once fixated (see also Kunar et al., 2017a, 2017b, 2020). As the mammograms were selected at random from DDSM the breast tissue varied in density. This affected target saliency from trial to trial (i.e. a cancer is likely to be more salient on less dense breast tissue). Please note this variation in target salience occurred across all of the experimental factors (i.e. target present vs absent trials and all the different CAD conditions). Furthermore, it replicates the high variability observed in clinical mammograms where saliency of the cancer is varied depending on breast tissue density and appearance of the cancerous mass. The CAD cues were the outline of a red box that subtended 1.1 degrees by 1.1 degrees at a viewing distance of 57 cm. All mammogram images were created offline.

**Fig. 1:**
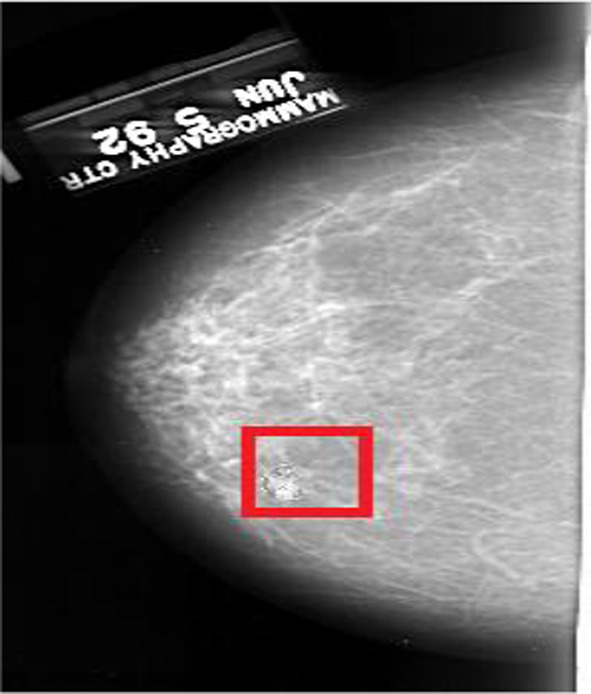
An example of a correct CAD trial in which the target was present. Here the CAD cue highlighted the presence of a cancer. In present, incorrect CAD trials the cancer appeared outside of the CAD cue and in present, no CAD trials a cancer was present but no CAD cue was shown

In each condition, there were 900 target absent trials and 100 target present trials (to give an overall target prevalence rate of 10%). For the target absent trials, 675 trials (75%) were presented without any CAD cues (correct CAD). The other 225 trials (25%) of target absent trials contained a CAD cue placed on a random area of the mammogram (incorrect CAD, see also Russell & Kunar, 2012; Kunar et al., 2017a, 2017b for similar methodology). For target present trials, 60 trials showed a CAD cue that correctly highlighted a mass (correct CAD), 20 trials showed a mass that fell outside of the CAD cue, with the CAD placed on another random area within the breast tissue (incorrect CAD) and 20 trials contained a mass but did not show any CAD cue (no CAD). Participants were aware that the target, if present, was likely to be cued by the CAD prompt, however, they were also told that on some trials there would be no CAD prompts on present trials, or the target could appear outside the CAD cue. Please note, that target present trials were more likely to contain a CAD cue than target absent trials (i.e. on 80% of trials vs 25% of trials) as in the field the CAD algorithms used would be more likely to display a prompt when a cancer is present than when it is absent. For each condition, participants viewed all 1000 mammogram images presented in a random order. An example image can be found in Fig. 1.

Participants completed two experimental conditions: an automatic CAD condition and an interactive CAD condition. For the automatic condition participants were first shown a blank screen for 500 ms. They were then presented with one of the mammogram images. CAD cues were automatically presented at the same time as the mammogram. Participants were asked to judge whether the mass was present or absent by pressing either the ‘m’ or the ‘z’ key, respectively. If no response was made within 30 s the trial ‘timed-out’ and the next trial started automatically. Following a response or ‘time-out’, a blank screen was again displayed before the next fixation dot and trial. The interactive condition was similar, except that mammograms were first presented without CAD. Participants made an initial response as to whether a cancer was present or not by pressing the ‘m’ or ‘z’ key, respectively. They were then shown the next screen asking them “Do you want to check with the use of CAD?” They pressed the ‘y’ key if the answer was yes or the ‘n’ key if the answer was no. If they chose yes, the mammogram was re-presented with the CAD cue overlaid. If there was no CAD cue associated with that particular trial then the mammogram would be re-presented without any CAD cue. Participants were then again asked to respond as to whether a cancer was present or not by pressing ‘m’ or ‘z’, respectively. Participants were free to change their response from their initial response should they wish to. In each condition reaction times and error rates for both the initial responses (in the automatic and interactive condition) and confirm responses (in the interactive condition) were recorded. If participants chose not to see the CAD cue the experiment moved to the next trial.

Alongside the confirm response in the interactive condition, in both the automatic and the interactive conditions participants had the option of correcting their responses. If the participants recognized that they had made an error, they were able to correct it on the following trial, by pressing the ‘Escape’ key during any time of the next trial (see Fleck & Mitroff, 1997; Van Wert et al., 2009; Kunar et al., 2010, 2017a, 2017b, 2020, Russell & Kunar, 2012; Rich et al., 2008, for similar methodologies). This would log in the data file that the participant had noticed their mistake so that motor errors could be calculated. They then proceeded with the current trial as normal, responding with an ‘m’ or ‘z’ key if the target was present or absent, respectively. No feedback was given after any response, or correction, was made.

To familiarise themselves with the stimuli, participants were shown examples of the mammogram images and cancers prior to each of the experiments. In this training session participants were first shown images of the cancerous masses on their own. The experimenter gave participants information of what to look for (e.g. the cancers have a spiculated appearance). They were then shown 12 different mammograms, one after the other, each containing a cancer. Participants were asked to point to the cancer, while the experimenter was in the same room (the experimenter would provide feedback if needed). Once participants completed this cancer identification task and both the participant and experimenter were confident that the participant could identify a mass, they then proceeded to take a practice block before each experimental block. During this practice block the experimenter again ensured that participants were able to recognise the cancer, when present. If any of the participants had difficulties identifying the cancer they were shown more examples and could repeat the practice condition until both the participant and experimenter were confident that they were able to identify the cancer. However, all the participants responded correctly in the first practice session and none were asked to repeat it. RTs, self-corrections and error rates were recorded. Within each condition breaks occurred automatically every 200 trials, after which participants continued with the experiment when they were ready. Given the length of each condition, the automatic and interactive conditions took place over two different sessions, each lasting approximately 2 h. The presentation order of conditions was counterbalanced across participants.

As the results of interest are from cognitive rather than motor response errors (i.e. those that can be corrected in the field) the analyses were conducted using the self-corrected data (see also Kunar et al., 2017a, 2017b, 2020). RTs responded after 30 s and before 200 ms were considered outliers and removed from data analysis. Bayes Factors analyses were also reported (calculated with a Cauchy prior width of 0.707 using JASP version 0.9.2),^2^ alongside frequentist statistics. The inclusion of Bayesian analyses gave the advantage of being able to evaluate evidence in support of the null hypothesis (Wagenmakers et al., 2018a). The recommendations of Jeffreys (1961) were adopted, in which a BF_10_ (which compares evidence of the alternative hypothesis to evidence for the null hypothesis) of 1 to 3 provides *anecdotal* evidence for the alternative, a BF_10_ of 3 to 10 provides *substantial* evidence for the alternative, a BF_10_ of 10 to 30 provides *strong* evidence for the alternative, a BF_10_ of 30 to 100 provides *very strong* evidence for the alternative and a BF_10_ of greater than 100 provides *decisive* evidence for the alternative. The inverse of these numbers (BF_01_) provide evidence in support the null hypothesis (Jarosz & Wiley, 2014).

## Results

**Fig. 2:**
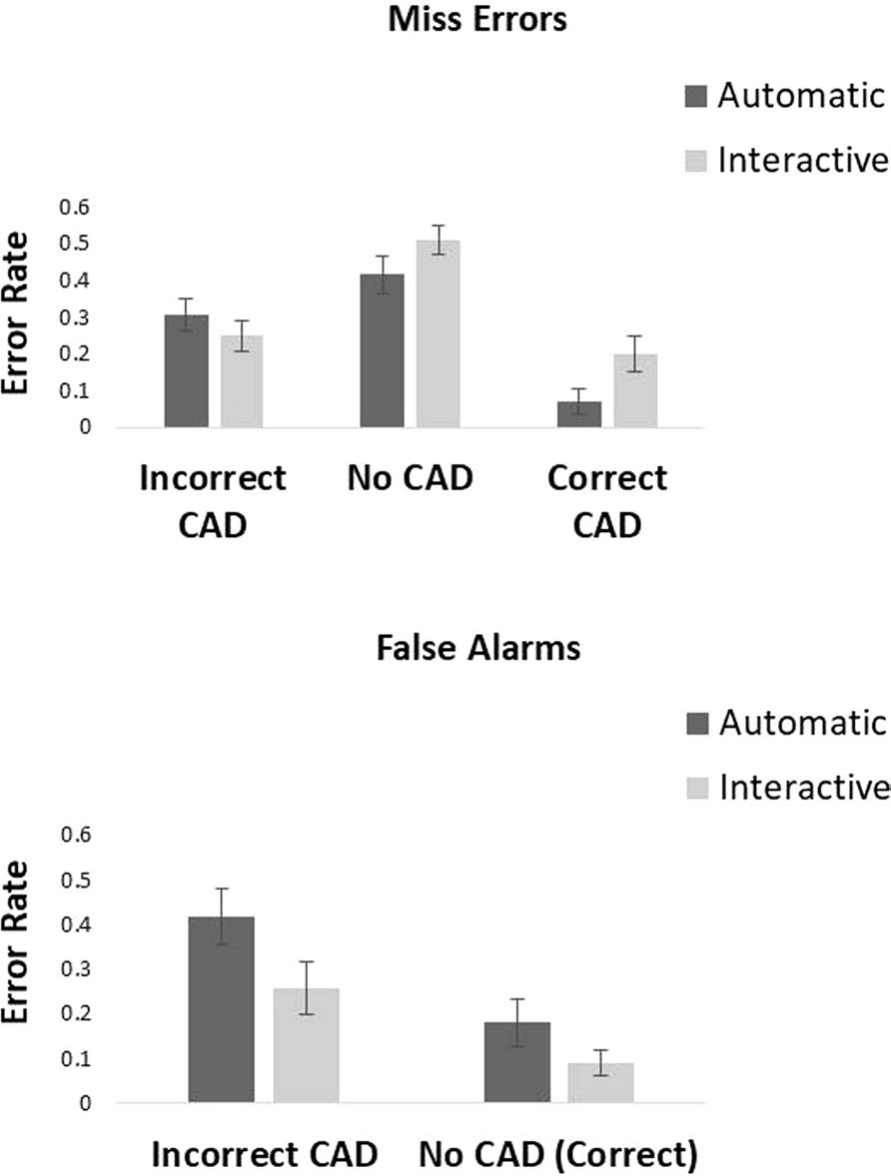
Proportion of miss errors and false alarms in the automatic and interactive conditions of Experiment 1

**Fig. 3:**
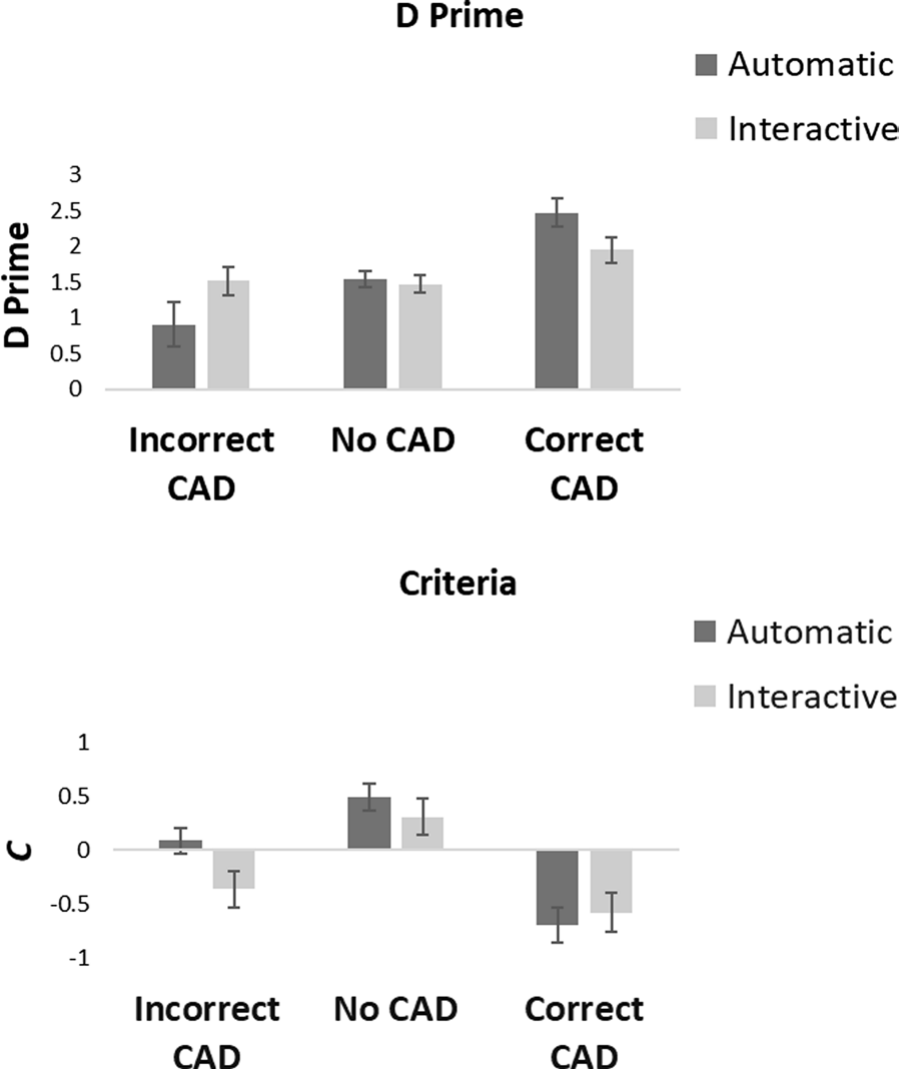
*D*′ and *c* values for the automatic and interactive conditions in Experiment 1

One participant was removed from analysis as 82.5% of their RTs were faster than 200 ms in the interactive condition. For the other 19 participants 14.3% of all data were removed as outliers.^3^ Error rates and mean correct reaction times for all conditions are presented in Figs. 2 and 3.

The experiment investigated whether cancer detection was improved when participants could choose to interact with CAD compared to when CAD was presented automatically alongside the mammogram. Miss errors and false alarms in the interactive condition were calculated by the proportion of cancers detected at the ‘final’ response. This final response varied depending on whether participants had chosen to check CAD on a particular trial. For trials in which CAD was checked, the final response was the response following the presentation of CAD. For trials where participants chose not to check CAD, the final response was the response participants made before the trial moved onto the next display.

Participants chose to check CAD on 34.3% of all trials in the interactive condition. A *t*-test was used to investigate whether participants were more likely to choose to view CAD when a cancer was present in the display compared to when it was not. The results showed that there was no difference in whether participants chose to check CAD if the target was present (39.9% of trials) versus absent (34.5% of trials), *t*(18) = 1.11 *p* = 0.28, *d* = 0.26, with anecdotal evidence in support of the null, BF_10_ = 0.41.

### Miss errors

Looking at Fig. 2, we see that miss errors were higher overall in the interactive condition than the automatic condition. This was particularly the case when the CAD cue was correct. They were also affected by CAD cue. A 2 × 3 within-participants ANOVA on miss errors with factor of condition (automatic vs interactive CAD) and CAD (correct CAD, incorrect CAD and no CAD) showed there to be a significant main effect of condition, *F*(1, 18) = 5.13, *p* = 0.036, *η*_p_^2^ = 0.22 in which there were fewer miss errors in the automatic than the interactive condition. There was also a significant main effect of CAD, *F*(2, 36) = 65.04, *p* < 0.001, *η*_p_^2^ = 0.78, in which there were fewer miss errors in the correct CAD, followed by incorrect CAD and then the no CAD conditions. There was a significant condition × CAD interaction, *F*(2, 36) = 6.33, *p* = 0.004 *η*_p_^2^ = 0.26. Planned *t*-tests showed that with correct CAD there were fewer miss errors in the automatic compared to the interactive CAD condition, *t*(18) = 4.02, *p* < 0.001, *d* = 0.92, with very strong evidence in support of the alternative BF_10_ = 44.32. When CAD was incorrect there was no difference in miss errors between the automatic condition and the interactive CAD condition, *t*(18) = 1.72, *p* = 0.10, *d* = 0.40, with anecdotal evidence in support of the null, BF_10_ = 0.82. For no CAD trials there was also no difference in miss errors between the automatic and interactive condition, *t*(18) = 1.81, *p* = 0.09, *d* = 0.41, with anecdotal evidence in support of the null, BF_10_ = 0.93.

### False alarms

Looking at Fig. 2,^4^ we see that false alarms were higher overall in the automatic condition than the interactive condition. They were also affected by CAD cue. A 2 × 2 within-participants ANOVA on false alarms with factor of condition (automatic vs interactive CAD) and CAD (incorrect CAD, vs no CAD) showed there to be a main effect of condition, *F*(1, 18) = 15.88, *p* < 0.001, *η*_p_^2^ = 0.47, in which there were fewer false alarms in the interactive compared to the automatic condition. There was also a significant main effect of CAD, *F*(1, 18) = 26.46, *p* < 0.001, *η*_p_^2^ = 0.60, in which more false alarms were made in the incorrect CAD condition compared to the no CAD condition. The condition × CAD interaction was not significant, *F*(1, 18) = 1.29, *p* = 0.27 *η*_p_^2^ = 0.07. As the interaction was not significant the data were not analysed further.

### Signal detection theory analyses

Signal detection theory was used to calculate how CAD affected *d*′ (a change in sensitivity) and *c* (a change in criterion) across presentation conditions.^5^ Figure 3 shows the *d*′ and *c* values.

### Sensitivity (*d*′)

Figure 3 shows that although there was an effect of CAD on *d*′ there was little overall difference in *d*′ between the automatic and interactive conditions. A 2 × 3 within-participants ANOVA on *d*′ with factor of condition (automatic vs interactive CAD) and CAD (correct CAD, incorrect CAD and no CAD) showed there to be no main effect of condition, *F*(1, 18) = 5.39e−4, *p* = 0.98, *η*_p_^2^ = 2.99e−5. There was a significant main effect of CAD, *F*(2, 36) = 41.10, *p* < 0.001, *η*_p_^2^ = 0.70, in which *d*′ was greatest in the correct CAD, followed by the no CAD and then the incorrect CAD conditions. There was a significant condition × CAD interaction, *F*(2, 36) = 6.06, *p* = 0.005 *η*_p_^2^ = 0.25. Planned *t*-tests showed that with correct CAD there was no difference in *d*′ between the automatic and the interactive CAD condition,^6^*t*(18) = 1.85, *p* = 0.08, *d* = 0.42, with anecdotal evidence in support of the null, BF_10_ = 0.98. Neither was there a difference in *d*′ between automatic and interactive conditions when there was no CAD, *t*(18) = 0.41, *p* = 0.69, *d* = 0.09, with substantial evidence in support of the null, BF_10_ = 0.26, or when CAD was incorrect, *t*(18) = 1.56, *p* = 0.14, *d* = 0.36, with anecdotal evidence in support of the null, BF_10_ = 0.67.

### Criterion (*c*)

Figure 3 shows that criterion was affected both by CAD and by whether CAD was presented automatically or interactively. A 2 × 3 within-participants ANOVA on *c* with factor of condition (automatic vs interactive CAD) and CAD (correct CAD, incorrect CAD and no CAD) showed there to be a main effect of condition, *F*(1, 18) = 4.32, *p* = 0.05, *η*_p_^2^ = 0.19, in which *c* was greater in the automatic condition compared to the interactive condition, and a main effect of CAD, *F*(2, 36) = 70.46, *p* < 0.001, *η*_p_^2^ = 0.80, in which *c* was greatest in the no CAD condition followed by the incorrect CAD and then correct CAD conditions. The condition × CAD interaction was also significant, *F*(2, 36) = 4.88, *p* = 0.01 *η*_p_^2^ = 0.21. Planned *t*-tests showed that there was no difference in *c* between the automatic and interactive conditions when CAD was correct, *t*(18) = 0.73, *p* = 0.48, *d* = 0.17, with substantial evidence in support of the null, BF_10_ = 0.30, or when there was no CAD, *t*(18) = 1.62, *p* = 0.12, *d* = 0.37, with anecdotal evidence in support of the null, BF_10_ = 0.72. However, *c* was greater in the automatic than the interactive condition when CAD was incorrect, *t*(18) = 3.65, *p* = 0.002, *d* = 0.84, with strong evidence in support of the alternative BF_10_ = 22.0.

### Automatic versus checked interactive CAD

**Fig. 4:**
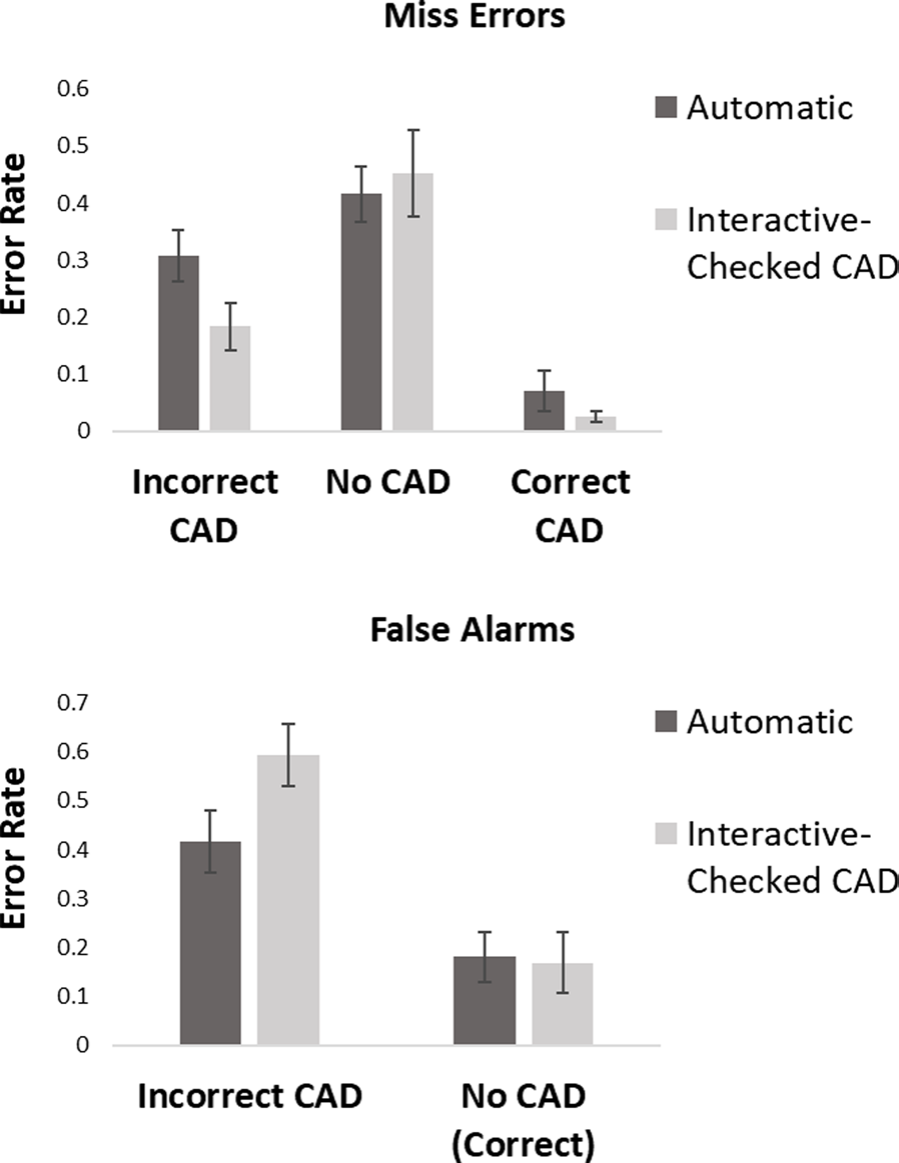
Proportion of miss errors and false alarms in the automatic and interactive checked CAD conditions of Experiment 1

As mentioned above, participants only chose to check CAD in the interactive condition on 34% of trials. To examine, how participants responded in the interactive condition when they chose to check CAD, error rates from these trials were compared to those of the automatic condition (see Fig. 4).^7^

### Miss errors: automatic versus interactive when CAD was chosen

Figure 4 shows that there was an effect of CAD on miss errors, however little difference in miss errors between the automatic and interactive checked-CAD condition. A 2 × 3 within-participants ANOVA on miss errors with factor of condition (automatic vs interactive checked-CAD) and CAD (correct CAD, incorrect CAD and no CAD) showed there to be no significant main effect of condition, *F*(1, 16) = 0.36, *p* = 0.56, *η*_p_^2^ = 0.02. There was a significant main effect of CAD, *F*(2, 32) = 52.54, *p* < 0.001, *η*_p_^2^ = 0.77, in which there were fewer miss errors in the correct CAD, followed by incorrect CAD and then the no CAD conditions. There was no significant condition × CAD interaction, *F*(2, 32) = 1.88, *p* = 0.17, *η*_p_^2^ = 0.11. As the interaction was not significant the data were not analysed further.

### False alarms: automatic versus interactive when CAD was checked

Figure 4 shows that there was an effect of CAD on false alarms. There was no difference in false alarms between presentation mode when there was no CAD cue, however, there were more false alarms in the interactive CAD-checked condition than the automatic when CAD was incorrect. A 2 × 2 within-participants ANOVA on false alarms with factor of condition (automatic vs interactive checked-CAD) and CAD (incorrect CAD, vs no CAD) showed there to be no main effect of condition, *F*(1, 17) = 1.24, *p* = 0.28, *η*_p_^2^ = 0.07. There was a significant main effect of CAD, *F*(1, 17) = 45.42, *p* < 0.001, *η*_p_^2^ = 0.73, in which more false alarms were made in the incorrect CAD condition compared to the no CAD condition. The condition × CAD interaction was significant, *F*(1, 17) = 5.03, *p* = 0.04 *η*_p_^2^ = 0.23. Planned *t*-tests showed that when CAD was incorrect, a greater number of false alarms were made in the interactive checked-CAD condition than the automatic condition, *t*(17) = 2.60, *p* = 0.02, *d* = 0.61, with substantial evidence in support of the alternative, BF_10_ = 3.15. There was no difference in false alarms between the automatic and interactive checked-CAD condition, when no CAD cue was presented, *t*(18) = 0.14, *p* = 0.89, *d* = 0.03, with substantial evidence in support of the null, BF_10_ = 0.24.

## Discussion

Experiment 1 compared whether presenting CAD alongside the mammogram (in the automatic condition) led to better search at low prevalence than when participants could choose to interact with CAD. The results showed that overall, people made fewer miss errors in the automatic condition compared to the interactive condition (26% vs 32%, respectively). However, they also made more false alarm errors in the automatic condition compared to the interactive (30% vs 17%, respectively). The results are mixed in terms of outcomes. In terms of cancer detection, the automatic condition showed superior performance. In terms of minimising false alarms, the interactive condition was the better presentation mode.

Overall, the data from both conditions replicate the over-reliance pattern observed in previous work (e.g. Kunar et al., 2017a, 2017b; Russell & Kunar, 2012). Miss errors were reduced when the CAD cue was correct. However, when the CAD cue was incorrect or there was no CAD cue then miss errors were high. False alarms were also increased with the presence of an incorrect CAD cue. In all conditions having a correct CAD cue aided target detection and having an incorrect CAD cue led to poorer search performance. However, these effects on miss errors and false alarms were differentially mitigated by how the CAD cues were presented.

Examining the miss errors, the results showed that, when CAD was accurate search was better overall in the automatic than the interactive condition. As the CAD cue was highly salient, then under conditions where it was visible and correct, there would be an expected benefit of it being presented. As participants only chose to view the CAD cue on 34% of trials in the interactive condition, it makes sense that more targets were found in the automatic condition, given that CAD was utilised on all trials. Furthermore, the automatic condition showed no miss error *cost* in comparison to the interactive condition when CAD was either incorrect or not shown. In terms of cancer detection rates, the automatic condition is the most beneficial presentation mode.

However, examining the false alarms, there were a greater proportion of false alarms in the automatic condition compared to the interactive condition. This occurred for both incorrect and no CAD (correct) conditions. In a clinical setting, an increase in false alarms would manifest as an increase in the number of women that are falsely recalled for further tests. This has serious financial and psychological implications for the women involved (Aro, 2000). Having the CAD cue be interactive mitigates these costs, but with the caveat that, overall, more women go undiagnosed as having a mass.

Unsurprisingly, CAD had an effect on sensitivity (as measured by *d*′) with an increase in sensitivity to detect a target when the CAD cue was correct in comparison to the no CAD and incorrect CAD condition. Although there was a trend for sensitivity to be lower in the interactive condition for correct CAD trials, (which also corresponds with the increase in miss errors for these trials), there was no overall difference in sensitivity when *d*′ was compared across automatic or interactive conditions. When examining criteria, CAD had an effect on response criteria with a shift to a more liberal response criteria in the correct CAD condition. Interestingly, there was an overall shift in response criteria between presentation modes with the interactive condition showing a more liberal response bias than the automatic. This shift in response bias was likely to be driven by the incorrect CAD condition. Interestingly, there was no clear effect of this response bias on the miss errors or false alarms when the interactive CAD trials were examined, as a whole. However, looking at the error rates in Fig. 4, we see that false alarms were higher in the interactive-Checked CAD condition than the automatic.^8^ This increase in false alarms is consistent with a more liberal response bias, where participants required less evidence to respond that a target is present.

The data are also of interest when we examine the proportion of times that participants checked CAD in the interactive condition. Participants only checked CAD on 34% of trials. This is far from ideal given the premise that CAD is to act as a ‘second reader’ in place of a radiologist. CAD can only be effective if it is chosen to be used as a tool to help search. If readers instead chose not to use CAD in favour of reading the mammograms alone this limits the efficacy of CAD technology. We discuss this further in the General Discussion.

Experiment 1 examined how the presentation of CAD affected peoples’ search performance at low prevalence. Participants either viewed the CAD cues simultaneously with the mammogram or could use them interactively should they wish, as a tool to confirm their response. The miss error data contradict the prediction that there should be no difference in miss errors when the CAD cue was correct. However, as mentioned above in this experiment participants only chose to use the CAD cues on 34% of the trials. Therefore, for the majority of trials in the interactive condition participants chose not to view the CAD cue. Experiment 2 investigates whether a similar pattern of results occurs on trials where participants were always shown the CAD cue, after they had searched the mammogram without CAD initially. This was again compared to an automatic condition, where the CAD cues were automatically shown to participants on initial presentation of the mammogram.

## Experiment 2

### Method

#### Participants

Twenty participants (*M* = 19.5 years, 14 female, 6 male) took part in Experiment 2. All participants had normal or corrected-to-normal vision. Participants were recruited separately from Experiment 1, however, they were not excluded from participation had they already taken part in Experiment 1.^9^

#### Stimuli and procedure

Participants completed two conditions: an *Automatic* condition and a *Confirm* CAD condition. The stimuli and procedure for the automatic condition were identical to those used in Experiment 1. The confirm condition was similar to the interactive condition of Experiment 1, except that after viewing and responding to the initial mammogram on each trial, participants were always shown the CAD cues. If there was no CAD cue associated with that particular trial, then the mammogram would be re-presented without the CAD cue. Participants were then asked to give a second response as to whether a cancer was present by pressing an ‘m’ if the cancer was present or a ‘z’ if the cancer was absent. The second response could either confirm or change their initial response. Once the second response had been made the next trial began. RTs and errors were recorded after both responses. Given the high proportion of trials where people responded faster than 200 ms in Experiment 1, we encouraged people to make sure they took time to search the display before response in all trials, in Experiment 2. Similar to Experiment 1, participants took part in the automatic and confirm conditions over two different sessions, each lasting approximately 2 h. The presentation order of conditions was counterbalanced across participants.

## Results

**Fig. 5:**
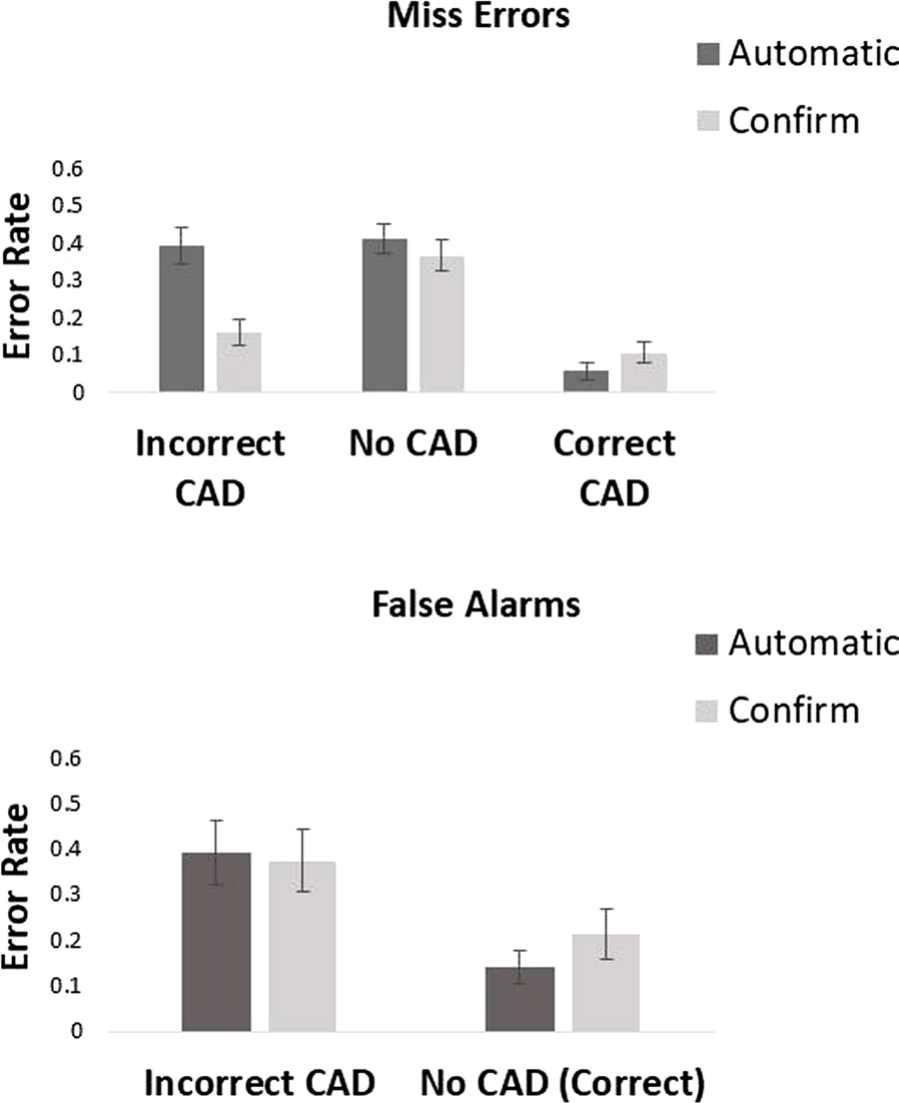
Proportion of miss errors and false alarms in the automatic and confirm conditions of Experiment 2

**Fig. 6:**
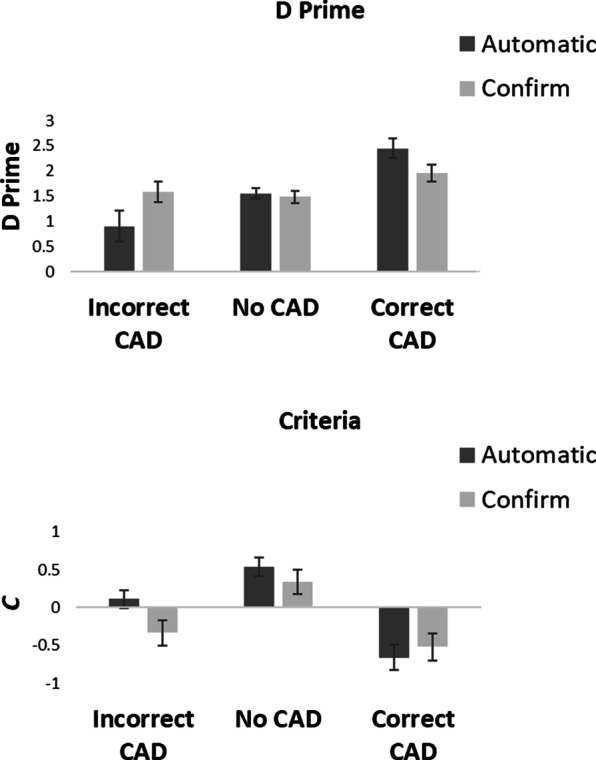
*D*′ and *c* values for the automatic and confirm conditions in Experiment 2

Due to a programming error some participants only had a time-out period of 10 s (rather than 30 s). To rectify this, and as most people responded within this time period, we removed all trials where participants took longer to respond than 10 s from analysis. Trials where participants responded faster than 200 ms were also removed from analysis. In total, these outliers led to the removal of 1.1% of all data. Error rates and mean correct reaction times for all conditions are presented in Figs. 5 and 6.

### Miss errors

Looking at Fig. 5, we see that miss errors were lower overall in the confirm condition than the automatic condition. This was particularly the case when the CAD cue was incorrect. They were also affected by CAD cue. A 2 × 3 within-participants ANOVA on miss errors with factor of condition (automatic vs confirm CAD) and CAD (correct CAD, incorrect CAD and no CAD) showed there to be a significant main effect of condition, *F*(1, 19) = 11.43, *p* = 0.003, *η*_p_^2^ = 0.38, in which overall there were fewer miss errors in the confirm condition than the automatic condition. There was also a main effect of CAD, *F*(2, 38) = 52.42, *p* < 0.001, *η*_p_^2^ = 0.73, in which there were fewer miss errors in the correct CAD, followed by incorrect CAD and then the no CAD conditions. There was a significant condition × CAD interaction, *F*(2, 38) = 22.15, *p* < 0.001 *η*_p_^2^ = 0.54. Planned *t*-tests showed that participants made fewer miss errors in the automatic than the confirm CAD condition when the CAD cue was correct, *t*(19) = 3.19, *p* = 0.005, *d* = 0.71 with substantial evidence in support of the alternative BF_10_ = 9.39. However, when the CAD cue was incorrect participants missed more targets in the automatic condition than in the confirm CAD condition, *t*(19) = 4.90, *p* < 0.001, *d* = 1.10, with decisive evidence in support of the alternative, BF_10_ = 277.75. There was no difference in miss errors across conditions when the target was present with no CAD cue, *t*(19) = 1.55, *p* = 0.14, *d* = 0.35, with anecdotal evidence in support of the null, BF_10_ = 0.65.

### False alarms

Looking at Fig. 5, we see that there was little effect of presentation mode on false alarms. However, false alarms were affected by the CAD cue. A 2 × 2 within-participants ANOVA on false alarms with factor of condition (automatic vs confirm CAD) and CAD (incorrect CAD vs no CAD) showed there to be no main effect of condition, *F*(1, 19) = 0.53, *p* = 0.48, *η*_p_^2^ = 0.03. However, there was a significant main effect of CAD, *F*(1, 19) = 16.52, *p* < 0.001, *η*_p_^2^ = 0.47, in which more false alarms were made in the incorrect CAD compared to the no CAD condition. There was also a significant condition × CAD interaction, *F*(1, 19) = 6.19, *p* = 0.02 *η*_p_^2^ = 0.25. Planned *t*-tests showed there was no significant difference in false alarms between the automatic and confirm CAD conditions when the incorrect CAD cue was shown, *t*(19) = 0.48, *p* = 0.64, *d* = 0.11, with substantial evidence in support of the null, BF_10_ = 0.26. Neither was there a significant difference in false alarms between conditions when no CAD cue was present, *t*(19) = 1.83, *p* = 0.08, *d* = 0.41, with anecdotal evidence in support of the null, BF_10_ = 0.93.

### Signal detection theory analyses

Signal Detection Theory was used to calculate how CAD affected *d*′ (a change in sensitivity) and *c* (a change in criterion) across presentation conditions. Figure 6 shows the *d*′ and *c* values.

### Sensitivity (*d*′)

Figure 6 shows that although there was an effect of CAD on *d*′ there was little overall difference in *d*′ between the automatic and confirm conditions. A 2 × 3 within-participants ANOVA on *d*′ with factor of condition (automatic vs confirm CAD) and CAD (correct CAD, incorrect CAD and no CAD) showed there to be no main effect of condition, *F*(1, 19) = 0.04, *p* = 0.85, *η*_p_^2^ = 0.002. There was a significant main effect of CAD, *F*(2, 38) = 39.73, *p* < 0.001, *η*_p_^2^ = 0.68, in which *d*′ was greatest in the correct CAD, followed by the no CAD and then the incorrect CAD conditions. There was a significant condition × CAD interaction, *F*(2, 38) = 7.14, *p* = 0.002 *η*_p_^2^ = 0.27. Planned *t*-tests showed that with correct CAD there was no difference in *d*′ between the automatic and the confirm CAD condition, *t*(19) = 1.82, *p* = 0.09, *d* = 0.41, with anecdotal evidence in support of the null BF_10_ = 0.92. Neither was there a difference in *d*′ between automatic and confirm conditions when there was no CAD, *t*(19) = 0.42, *p* = 0.68, *d* = 0.09, with substantial evidence in support of the null, BF_10_ = 0.25, or when CAD was incorrect, *t*(19) = 1.81, *p* = 0.09, *d* = 0.40, with anecdotal evidence in support of the null, BF_10_ = 0.91.

### Criterion (*c*)

Figure 6 shows that criterion was affected both by CAD and by whether CAD was presented in automatic or confirm mode. A 2 × 3 within-participants ANOVA on *c* with factor of condition (automatic vs confirm CAD) and CAD (correct CAD, incorrect CAD and no CAD) showed there to be a main effect of condition, *F*(1, 19) = 4.35, *p* = 0.05, *η*_p_^2^ = 0.19, in which *c* was greater in the automatic condition compared to the confirm condition and a main effect of CAD, *F*(2, 38) = 74.20, *p* < 0.001, *η*_p_^2^ = 0.80, in which *c* was greatest in the no CAD condition followed by the incorrect CAD and then correct CAD conditions. The condition × CAD interaction was also significant, *F*(2, 38) = 5.76, *p* = 0.007 *η*_p_^2^ = 0.23. Planned *t*-tests showed that there was no difference in *c* between the automatic and confirm conditions when CAD was correct, *t*(19) = 0.92, *p* = 0.37, *d* = 0.21, with substantial evidence in support of the null, BF_10_ = 0.34, or when there was no CAD, *t*(19) = 1.76, *p* = 0.10, *d* = 0.39, with anecdotal evidence in support of the null, BF_10_ = 0.85. However, *c* was greater in the automatic than the confirm condition when CAD was incorrect, *t*(19) = 3.82, *p* = 0.001, *d* = 0.85, with very strong evidence in support of the alternative BF_10_ = 31.93.

### Comparison of interactive versus confirm conditions

Given that we are interested in how presentation modes of CAD affect miss errors and false alarm rates, two separate ANOVAs were run to compare responses across the interactive condition of Experiment 1, with the confirm condition of Experiment 2.

### Miss errors

There were fewer miss errors overall in the confirm compared to the interactive condition. There was also an effect of CAD. A 2 × 3 ANOVA on miss errors with a between-participant factor of condition (interactive vs confirm CAD) and within-participant factor of CAD (correct CAD, incorrect CAD and no CAD) showed there to be a significant main effect of condition, *F*(1, 37) = 5.25, *p* = 0.03, *η*_p_^2^ = 0.12, in which overall there were fewer miss errors in the confirm condition than the interactive condition. There was also a main effect of CAD, *F*(2, 74) = 66.49, *p* < 0.001, *η*_p_^2^ = 0.64, in which there were fewer miss errors in the correct CAD, followed by incorrect CAD and then the no CAD conditions. There was no significant condition × CAD interaction, *F*(2, 74) = 0.69, *p* = 0.51 *η*_p_^2^ = 0.02. As the interaction was not significant the data were not analysed further.

### False alarms

Although there was an effect of CAD on false alarms there was no effect of presentation mode. A 2 × 2 ANOVA on false alarms with a between-participant factor of condition (interactive vs confirm CAD) and CAD (incorrect CAD vs no CAD) showed there to be no main effect of condition, *F*(1, 37) = 3.12, *p* = 0.09, *η*_p_^2^ = 0.08. However, there was a significant main effect of CAD, *F*(1, 37) = 18.64, *p* < 0.001, *η*_p_^2^ = 0.34, in which more false alarms were made in the incorrect CAD compared to the no CAD condition. There was no significant condition × CAD interaction, *F*(1, 37) = 0.01, *p* = 0.93 *η*_p_^2^ = 2.06e−4. As the interaction was not significant the data were not analysed further.

## Discussion

Experiment 2 compared an automatic condition, to a confirm condition. The results showed that the confirm presentation mode showed better performance than the automatic presentation mode, in terms of fewer miss errors and no cost to false alarms. A between-experiment comparison further showed that there was an overall benefit of the confirm mode in comparison to the interactive mode in terms of fewer miss errors (21% vs 32%, respectively) and no cost in terms of false alarms.

Overall, the results from the automatic condition again indicate an over-reliance on the CAD cue, replicating data from Experiment 1 and Kunar et al. (2017a, 2017b). Miss errors were greatly reduced if the mass appeared within the CAD cue. However, if the mass appeared outside of the CAD cue or no CAD was shown then errors were much higher. Furthermore, there were a greater number of false alarms when the CAD cue was incorrectly presented on target absent trials. People’s search performance was being adversely affected by incorrect CAD cues. Of importance, similar to Experiment 1, the cost of CAD was mitigated by how it was presented.

Unlike Experiment 1, there was no difference in false alarms between conditions. That is, neither the automatic of confirm presentation mode showed a benefit or a cost over the other in terms of false alarms. Examining miss errors, across all trials, fewer targets were missed in the confirm condition compared to the automatic condition (21% vs 29%). However, this difference in miss errors varied depending on the accuracy of the CAD cue. When CAD was accurate, more targets were missed in the confirm condition than the automatic condition. This is surprising, given that participants saw the CAD cue on *all* trials in *both* conditions. There was no difference in miss errors on trials when the CAD cue was not presented. However, on trials when the CAD cue highlighted an incorrect area, fewer miss errors were observed in the confirm compared to the automatic condition. There are two reasons why this might be. First, it could be that the presence of the CAD cue led participants to re-search the display more thoroughly when they were asked to confirm. To investigate this RTs were compared for participants initial response (without CAD) in the confirm condition to their second response, after they had been shown CAD. However, the results showed that RTs were significantly shorter after the second response when CAD had been presented, (1253 ms vs 519 ms respectively, *t*(19) = 7.40, *p* < 0.01, *d* = 1.65, with decisive evidence for the alternative, BF_10_ = 32,359.48). This suggests that participants were not taking the time to search the display thoroughly when they were being asked to confirm their response. Instead, it is more likely that participants believed the CAD algorithm had detected an anomaly that they have missed. In this case, they may be more likely to trust in the CAD cue versus their own judgement, following the over-reliance hypothesis.

Examining the SDT analyses, similar to Experiment 1, CAD had an effect on sensitivity (as measured by *d*′) with an increase in sensitivity to detect a target when the CAD cue was correct in comparison to the no CAD and incorrect CAD condition. However, there was no overall difference in sensitivity when *d*′ was compared across automatic and confirm conditions (although similar to Experiment 1, there was a trend for sensitivity to be lower in the confirm condition for correct CAD trials). When examining criteria, CAD had an effect on response criteria with a shift to a more liberal response in the correct CAD condition than in the incorrect and no CAD conditions. Participants also showed an overall difference in response bias between presentation conditions, where criteria in the confirm condition showed a more liberal response bias than in the automatic condition. Please note, that the SDT results are puzzling in relation to the overall error data. In terms of SDT, lower miss errors in the confirm condition would be thought to occur with a change in response bias and/or an increase in sensitivity. Although there was a change in response bias, which may have explained why participants missed fewer targets, this should also have resulted in an increase in false alarms. Furthermore, the *d*′ data showed no overall change in sensitivity. One reason for these differences may be that the decrease in confirm miss errors looks to be largely driven by the incorrect CAD cues. This may explain the results, as *d*′ in these trials showed a hint of change in which *d*′ was marginally greater in the confirm than in the automatic conditions. Further research would be needed to confirm this.

## General discussion

The work in this paper investigated how search for an LP target was affected by CAD presentation. Experiment 1 compared an automatic condition where CAD cues were presented simultaneously alongside a mammogram to an interactive condition, where participants chose whether or not to check CAD, after initial reading. Experiment 2 compared an automatic condition to a confirm condition, where participants first searched the display without CAD, before being shown the CAD cues on all trials and asked to confirm or change their response.

Overall, the data suggest that presenting CAD in confirm mode led to better search performance. Both in comparison to the automatic condition and the interactive condition, there were fewer miss errors in total and no cost in terms of false alarm rates. Du-Crow et al. (2019) suggested that the confirm presentation method was flawed as it gave readers a ‘safety net’ so that participants would be less likely to search the initial display thoroughly. However, the current results suggest otherwise, with search performance, in terms of finding cancers superior in the confirm condition, with little cost to false alarms.

Despite the overall benefit to search performance in the confirm condition, there was a small cost to miss errors when the CAD cue was correct. Miss errors for correct CAD conditions were higher in the confirm condition compared to the automatic condition (11% vs 6%, respectively). One potential reason may be that participants found it easier to over-ride the salient CAD cue if there was a delay to its onset with the original mammogram display. There is evidence to suggest that top-down attentional guidance mechanisms can increase with display time (Kunar et al., 2008; Watson & Humphreys, 1997), which may have allowed participants to better disregard the salient cue if it appeared later. Furthermore, presenting the CAD cue with the initial mammogram gave a strong exogenous signal that would result in high activation on a bottom-up saliency or priority map, making it hard to ignore (Itti and Koch, 2001; Wolfe, 2021). This salience may have affected people’s decisions so that they were more likely to indicate the presence of a target (Kunar et al., 2017a, 2017b). On the other hand, presenting the CAD cue after people have already made a judgement on target presence, may mitigate the salience of this cue as participants showed a confirmation bias for their initial decision. Confirmation bias has been shown to affect decision making in a number of medical environments (e.g. Croskerry, 2002; Pang et al., 2017; Tschan et al., 2009). Croskerry (2002) suggested that confirmation bias would lead to people disregarding important data if it disagrees with an initial medical decision. In terms of the current work this might mean that if the CAD cue opposed original judgement, participants may be more likely to dismiss it. Please note that this may have only happened in some trials. However, given that miss errors (and the variance) in CAD correct conditions were low, even a slight increase in miss errors would be enough to observe a significant difference. This may not be the case in conditions where miss errors and the variance was larger (e.g. false alarm trials). Although one could argue that the cost to miss errors was relatively small, the high health stakes of missing a cancer in a clinical environment ensure that it is important to keep miss errors to a minimum. This has implications, given that the confirm presentation mode is recommended by the FDA. Therefore, clinical readers should be advised of the importance of sufficient consideration of *all* CAD cues that are presented after initial reading, with potential referral to third parties or arbitration on cases where CAD cues are subsequently shown.

The above results suggest that the salience and presentation mode of CAD affects people’s judgements when it correctly cued the target. What about trials when the CAD cue was incorrect? This was important on false alarm trials, in situations where CAD incorrectly prompts an area. Across all conditions, false alarms were increased when CAD was presented on target- absent trials. Interestingly, there were no differences in the proportion of false alarms in the confirm and automatic conditions. However, false alarms were reduced overall, in the interactive condition in comparison to the automatic condition. There are two potential reasons for this. First, as mentioned above, it could be that again people were better able to disregard the salience of the CAD cue if there was a delay to its onset, giving participants a chance to read the mammogram first, without CAD. However, if this were the driving factor, in this case we would also expect a similar performance in the confirm condition. Second, given that participants only chose to check CAD on approximately a third of trials, there would have been many target-absent ‘incorrect CAD’ trials, in which CAD was not actually shown. In this case, participants’ judgements would not have been affected by the presence of the CAD cue. This second reason was supported when examining the false alarms in the interactive checked CAD trials, where false alarms increased when CAD cues were shown.

Our results suggest that there was little benefit of showing CAD interactively. This contrasts with the findings of Hupse et al. (2013) and Drew et al. (2020) who report benefits of interactive CAD. One reason is that Hupse et al (2013) used an enriched data set, with a high prevalent target. The results of this study again show the importance of the fact that prevalence rates of the target change search outcomes and needs to be considered when testing how best to use CAD systems. A second reason may be that in the current experiments, participants chose to use CAD on a relatively small proportion of trials in the interactive condition (i.e. on 34% of trials). The exact proportion of trials that CAD was used interactively for the work of Hupse et al. (2013) and Drew et al. (2020) is unknown. However, the studies by Drew et al. (2020) incentivised participants with point scoring for good search outcomes, thereby encouraging participants to make regular use of the interactive CAD. If participants were interacting with the CAD cues on the majority of trials then this may in practice look like the confirm presentation mode in the current work (which showed an overall improvement in search). It is likely that, the proportion of times that CAD is utilised affects search performance.

The uptake of CAD when readers can choose to use it is also an important point to consider. In this study, participants showed a limited use of CAD in the interactive condition. This is noteworthy, as it suggests a behavioural preference for participants to rely on their own judgements *without the use of CAD* for the majority of trials. Although they did not give an exact percentage, Hupse et al. (2013) also reported that readers showed a limited propensity to use the interactive CAD prompt. This is significant given that CAD has been proposed to act as a ‘second reader’ to replace double reading procedures (e.g. Azavedo et al., 2012). Double reading has been shown to be an effective method of reducing the LP effect and finding a rare cancer (e.g. Kunar et al., 2021). If participants chose not to engage with CAD, they are in effect implementing a sub-optimal single reading procedure, which would have implications in the clinical field. It will be up to future work to investigate the proportion of times that CAD is utilised in mammography under interactive conditions. However, if it is found that radiologists choose not to use CAD on the majority of trials then this highlights a separate failing in CAD technology in terms of user uptake.

The current work also replicated the over-reliance effect shown by Kunar et al. (2017a, 2017b). When the CAD cue was correct, people made fewer errors than when the CAD cue was incorrect, showing that people’s judgements, rightly and wrongly, were influenced by the presence of CAD. Interestingly, the confirm condition showed some evidence of mitigating the cost for miss errors, when the CAD cue was incorrect. In this condition miss errors were lower than in the automatic condition. It may be that having CAD as a ‘safety net’ is beneficial under these particular circumstances: participants were more likely to trust that CAD had detected a cancer that they had missed. Interestingly, in this particular instance, over-confidence in the technology led to an improvement in cancer detection.

Wolfe and Van Wert (2010) proposed a Multiple Decision Model to explain the high miss errors observed under LP, in which both the quitting threshold of search and the response bias changed at LP. The signal detection data of these experiments can also be used to help understand how CAD affects LP search, particularly when CAD cues are correct. In all experiments, the results showed that having a correct CAD leads to an improvement in sensitivity (as measured by *d*′). People were better able to detect a cancer, from a non-cancer, when it was prompted by CAD. There was also an effect of CAD on response criteria so that when CAD was correct participants required less evidence to respond to a target’s presence. This change in response bias mitigates the typical conservative shift observed in LP conditions (Wolfe and Van Wert, 2010; Horowitz, 2017; Drew et al., 2020; Kunar et al., 2021; Russell & Kunar, 2012; Van Wert et al., 2009; Wolfe & Van Wert, 2010). Both the change in sensitivity and response criteria led to a reduction in miss errors when CAD was correct. The SDT data can also be used to understand why the confirm condition produced the optimal search performance. Here we see that although the confirm condition did not show an overall improvement of sensitivity, it did lead to a change in response bias so that participants showed a more liberal response in accepting that a target was present. This change in response bias again mitigated the typical LP shift to a more conservative response proposed by the Multiple Decision Model (Wolfe and Van Wert, 2010).

The experiments in this study used a present/absent response task where participants pressed one key if they thought a target was present and another if the target was absent. For the purpose of these studies, on target present trials it was assumed that when people responded ‘target present’ they had found the cancer. However, it could be that sometimes participants were instead (incorrectly) responding to a ‘non-target’ area which they had falsely identified as a target. Future work should therefore consider using a localisation response, whereby participants clicked on the location of a target. This would help remove any ambiguity as to whether participants were responding correctly to the target or incorrectly to a non-target area.

Of final note, the results in this paper have implications for practice and policy in terms of CAD use and training of mammogram readers. Having CAD be presented in confirm mode should be the default recommendation for all clinical settings. However, it is also important to train mammogram readers as to how to best engage with this technology. For example, to mitigate errors due to confirmation bias and over-reliance effects, readers could be required to complete a training programme to educate them on cognitive and psychological factors that we now know influence medical search. This could include training on cognitive biases, decision-making, perception, prevalence and inattentional blindness (see Drew et al., 2013 for an example of how inattentional blindness affects search with medical images). Training courses have been offered as a solution to offset cognitive biases in other applied tasks (e.g. forensic psychology, Kassin et al., 2013) and provide a good way to educate readers on how to identify, and thus better avoid, cognitive ‘pitfalls’ that lead to inaccurate judgements. Furthermore, educational training has been found to be a highly effective method in combatting cognitive bias in applied settings (Sellier et al., 2019). These training courses would be particularly important for newly qualified radiologists given their lack of experience in mammography. Radiologists who are considered experts in their field and have acquired a vast range of experience may be more likely to disregard inaccurate CAD prompts and will have a greater experience in recognising a cancer when a CAD prompt fails. Therefore, CAD could disproportionately affect the judgements of radiologists and readers who are early on in their career. Targeting readers with less clinical experience as those eligible for training in cognitive bias and other psychological factors could be an effective method to help reduce medical errors.

## Data Availability

The datasets used and/or analysed during the current study are available from the corresponding author on reasonable request.

## Abbreviations

LP: Low prevalence
HP: High prevalence
CAD: Computer aided detection
FDA: Food and Drug Administration
ANOVA: Analysis of variance
RT: Reaction time
UK: United Kingdom
USA: United States of America
MDM: Multiple decision model

## Footnotes

1 Please note that some of the images from the DDSM contained dates and/or artefacts on the background of the image similar to images seen by radiologists in clinical mammography. However, as the dates/artefacts only appeared on the background of the image they did not affect the actual search task.

2 Please note we only report Bayes statistics for the planned *t*-tests as Bayes factors for repeated measures ANOVAs still has its challenges and is an ongoing topic of research (Wagenmakers et al., 2018b).

3 This is a higher proportion of outliers than typically observed in a visual search task. This high proportion was due to participants responding faster than 200 ms (resulting in 13.9% of outliers). These fast RTs were witnessed across participants. This point was addressed in Experiment 2 by encouraging participants to search the display thoroughly before response. Nevertheless, even with the removal of outliers in Experiment 1, this still led to 86% of data available for analysis.

4 Please note that in these experiments participants did not respond to the location of the cancer, rather they responded to whether they believed a target was present in the display. For the purpose of these experiments we consider target present trials, where participants pressed the target present key as a ‘hit’. However, it could be that on some trials participants had not found the actual cancer but were responding to a non-target area that they believed contained a mass. We discuss this further in the General Discussion.

5 False alarm or miss error rates of 0 and 1 were adjusted using the formulas 1/2*n* and 1 − (1/2*n*), where *n* = the number of trials (Macmillan & Kaplan, 1985, see also Russell & Kunar, 2012, Wolfe et al., 2007, and Kunar et al., 2021, who used this procedure).

6 In several of the analyses the p-values indicate that there was a trend towards a significant result (with a p-value between 0.05 and 0.1, Olsson-Collentine et al., 2019). For continuity and the purpose of the results, I state that these marginal values are not significant. However, they are of interest when examining the statistical interactions.

7 Please note that in some conditions participants never chose the option to check CAD, therefore participant numbers in the analyses vary depending on data availability.

8 Miss errors were also numerically lower in the interactive-checked CAD condition in comparison to the Automatic condition for Incorrect CAD cues, however this difference was not significant, *t*(16) = 1.61, *p* = 0.13.

9 As data were anonymised for ethical reasons it cannot be determined how many participants (if any) took part in both Experiments 1 and 2. Participants were not asked if they had completed any previous experiments conducted by the research group, so it is unknown how (or if) this affected the data. If participants took part in the previous study, their data may have been affected by learning effects. However, please note that even with any learning effects participants still showed an over-reliance of CAD in all conditions.
